# Supplementary material for: Motivators and barriers to mouthguard compliance by adult Gaelic football athletes
Source: PLoS One. 2025 Apr 23;20(4):e0315555. doi: 10.1371/journal.pone.0315555 (PMC12017504; doi:10.1371/journal.pone.0315555)
Supplement: S1 File — (DOCX) [file pone.0315555.s001.docx]

**Supplementary Material 1**

STROBE Statement—checklist of items that should be included in reports of observational studies

|  | Item No. | Recommendation | Page  No. | Relevant text from manuscript |
| --- | --- | --- | --- | --- |
| **Title and abstract** | 1 | (*a*) Indicate the study’s design with a commonly used term in the title or the abstract | 2 | Line 51 |
|  |  | (*b*) Provide in the abstract an informative and balanced summary of what was done and what was found | 2 | Lines 42-75 |
| Introduction | | | |  |
| Background/rationale | 2 | Explain the scientific background and rationale for the investigation being reported | 3 | Lines 91-142 |
| Objectives | 3 | State specific objectives, including any prespecified hypotheses | 3, 4 | Lines 136-142 |
| Methods | | | |  |
| Study design | 4 | Present key elements of study design early in the paper | 4 | Lines 147-148 |
| Setting | 5 | Describe the setting, locations, and relevant dates, including periods of recruitment, exposure, follow-up, and data collection | 4 | Lines 145-169 |
| Participants | 6 | (*a*) *Cohort study*—Give the eligibility criteria, and the sources and methods of selection of participants. Describe methods of follow-up  *Case-control study*—Give the eligibility criteria, and the sources and methods of case ascertainment and control selection. Give the rationale for the choice of cases and controls  *Cross-sectional study*—Give the eligibility criteria, and the sources and methods of selection of participants | 4 | Lines 150-153 |
|  |  | (*b*) *Cohort study*—For matched studies, give matching criteria and number of exposed and unexposed  *Case-control study*—For matched studies, give matching criteria and the number of controls per case |  |  |
| Variables | 7 | Clearly define all outcomes, exposures, predictors, potential confounders, and effect modifiers. Give diagnostic criteria, if applicable | 4 | Lines 162-169 |
| Data sources/ measurement | 8* | For each variable of interest, give sources of data and details of methods of assessment (measurement). Describe comparability of assessment methods if there is more than one group | 4 | Lines 162-182 |
| Bias | 9 | Describe any efforts to address potential sources of bias | 4 | Lines 157-159 |
| Study size | 10 | Explain how the study size was arrived at | 4 | Lines 155-156 |

Continued on next page

| Quantitative variables | 11 | Explain how quantitative variables were handled in the analyses. If applicable, describe which groupings were chosen and why | 4 | Lines 173-182 |
| --- | --- | --- | --- | --- |
| Statistical methods | 12 | (*a*) Describe all statistical methods, including those used to control for confounding | 4 | Lines 173-182 |
|  |  | (*b*) Describe any methods used to examine subgroups and interactions | 4 | Lines 173-182 |
|  |  | (*c*) Explain how missing data were addressed | 4 | Lines 157-159 |
|  |  | (*d*) *Cohort study*—If applicable, explain how loss to follow-up was addressed  *Case-control study*—If applicable, explain how matching of cases and controls was addressed  *Cross-sectional study*—If applicable, describe analytical methods taking account of sampling strategy | x | N/A |
|  |  | (*e*) Describe any sensitivity analyses |  |  |
| Results | | | | |
| Participants | 13* | (a) Report numbers of individuals at each stage of study—eg numbers potentially eligible, examined for eligibility, confirmed eligible, included in the study, completing follow-up, and analysed | 4 | Lines 156, 186 |
|  |  | (b) Give reasons for non-participation at each stage | 4 | Lines 157-158 |
|  |  | (c) Consider use of a flow diagram | x | N/A |
| Descriptive data | 14* | (a) Give characteristics of study participants (eg demographic, clinical, social) and information on exposures and potential confounders | 5-6 | Table 1 |
|  |  | (b) Indicate number of participants with missing data for each variable of interest | x | N/A |
|  |  | (c) *Cohort study*—Summarise follow-up time (eg, average and total amount) | x | N/A |
| Outcome data | 15* | *Cohort study*—Report numbers of outcome events or summary measures over time | x | N/A |
|  |  | *Case-control study—*Report numbers in each exposure category, or summary measures of exposure | x | N/A |
|  |  | *Cross-sectional study—*Report numbers of outcome events or summary measures | 6 | Table 2 |
| Main results | 16 | (*a*) Give unadjusted estimates and, if applicable, confounder-adjusted estimates and their precision (eg, 95% confidence interval). Make clear which confounders were adjusted for and why they were included | 6, 9 | Table 2, Table 3 |
|  |  | (*b*) Report category boundaries when continuous variables were categorized | 6, 9 | Table 2, Table 3 |
|  |  | (*c*) If relevant, consider translating estimates of relative risk into absolute risk for a meaningful time period | x | N/A |

Continued on next page

| Other analyses | 17 | Report other analyses done—eg analyses of subgroups and interactions, and sensitivity analyses | Supplementary Material | Supplementary Material |
| --- | --- | --- | --- | --- |
| Discussion | | | | |
| Key results | 18 | Summarise key results with reference to study objectives | 10 | Lines 278-339 |
| Limitations | 19 | Discuss limitations of the study, taking into account sources of potential bias or imprecision. Discuss both direction and magnitude of any potential bias | 11, 12 | Lines 364-378 |
| Interpretation | 20 | Give a cautious overall interpretation of results considering objectives, limitations, multiplicity of analyses, results from similar studies, and other relevant evidence | 10, 11 | Lines 278-360 |
| Generalisability | 21 | Discuss the generalisability (external validity) of the study results | 11 | Lines 343-260 |
| Other information | |  | | |
| Funding | 22 | Give the source of funding and the role of the funders for the present study and, if applicable, for the original study on which the present article is based | x | N/A |

*Give information separately for cases and controls in case-control studies and, if applicable, for exposed and unexposed groups in cohort and cross-sectional studies.

**Note:** An Explanation and Elaboration article discusses each checklist item and gives methodological background and published examples of transparent reporting. The STROBE checklist is best used in conjunction with this article (freely available on the Web sites of PLoS Medicine at http://www.plosmedicine.org/, Annals of Internal Medicine at http://www.annals.org/, and Epidemiology at http://www.epidem.com/). Information on the STROBE Initiative is available at www.strobe-statement.org.

**Supplementary Material 2**

**Anonymous Online Consent Form**

This is a research project being conducted by XX, XX and XX at XXX. You are invited to participate in this research project because you are a Gaelic Football athlete. Your participation in this research study is voluntary. You may choose not to participate. If you decide to participate in this research survey, you may withdraw at any time. If you decide not to participate in this study or if you withdraw from participating at any time, you will not be penalized. The procedure involves completing an online survey that will take approximately 5 minutes. Your responses will be confidential, and we do not collect identifying information such as your name, email address or IP address. All data is stored in a password protected electronic file. To help protect your confidentiality, the surveys will not contain information that will personally identify you. The results of this study will be used for scholarly purposes only and may be shared with XXX representatives. If you have any questions about the research study, please contact [redacted for blind review]

Before beginning the survey, please answer the following questions:

I have read the Plain Language Statement (or had it read to me)

- Yes
- No

I understand the information provided

- Yes
- No

I have had an opportunity to ask questions and discuss this study

- Yes
- No

I understand the information provided in relation to data protection

- Yes
- No

I have received satisfactory answers to all of my questions

- Yes
- No

I understand I may withdraw from the research study at any point

- Yes
- No

I have read and understand the arrangements to be made to protect confidentiality of data, including that confidentiality of information provided is subject to legal limitations

- Yes
- No

I have read and understand confirmations relating to any other relevant information as indicated in the Plain Language Statement

- Yes
- No

I consent to participate in this research study

- Yes
- No

**Online Survey**

**Section 1. Demographics**

Q.1. Gender (Please select one answer)

- Male
- Female
- Other:
- Prefer not to say

Q.2. Age

Open numerical response

Q.3. Tick the teams for which you currently play Gaelic football with. Please select all that apply.

- Senior Inter-County
- Underage Inter-County
- Senior Club
- Intermediate Club
- Junior Club
- Senior Collegiate
- Intermediate Collegiate
- Junior Collegiate
- Fresher Collegiate
- Underage Club

Q.4. How many years have you been playing Gaelic football for?

- < 1 year
- 1 year
- 2 years
- 3 years
- 4 years
- 5 years
- 6 years
- 7 years
- 8 years
- 9 years
- 10 years
- 11 years
- 12 years
- 13 years
- 14 years
- 15 years
- 16 years
- 17 years
- 18 years
- 19 years
- 20 years
- >20 years

**Section 2. Mouthguards**

It is mandatory for all players, from underage up to senior intercounty, to wear a mouthguard in all Gaelic football games and trainings.

Q.5. How would you rate the compliance of Gaelic football players in the use of mouthguards during training? Compliance can be defined as “action in accordance with rules and regulations”. Very poor compliance would suggest a small minority (e.g. <10%) acting in accordance with rules, while excellent compliance would suggest a large majority (e.g. >90%) acting in accordance with rules. Please select one answer.

- Very poor
- Poor
- Average
- Good
- Excellent

Q.6. How would you rate the compliance of Gaelic football players in the use of mouthguards during games? Compliance can be defined as “action in accordance with rules and regulations”. Very poor compliance would suggest a small minority (e.g. <10%) acting in accordance with rules, while excellent compliance would suggest a large majority (e.g. >90%) acting in accordance with rules. Please select one answer.

- Very poor
- Poor
- Average
- Good
- Excellent

Q.7. Referring to your own personal practices, do you wear a mouthguard when training? Please select one answer.

- Yes, always (100% of the time)
- Yes, more often than not (75% of the time)
- Yes, sometimes (50% of the time)
- Rarely, it’s always in my sock, and I put it in when instructed to do so (e.g. by management, referees, teammates)
- No, I never wear it during training

Q.8. Referring to your own personal practices, do you wear a mouthguard when playing matches? Please select one answer.

- Yes, always (100% of the time)
- Yes, more often than not (>75% of the time)
- Yes, sometimes (>50% of the time)
- Rarely, it’s always in my sock, and I put it in when instructed to do so (e.g. by management, referees, teammates)
- No, I never wear it during matches

Q.9. If you do wear a mouthguard, why do you wear one/ what are your motives for wearing one? Please rate the following answers: Strongly agree, agree, neutral, disagree, strongly disagree.

- It's the rules of the sport, I have to
- For injury insurance scheme cover
- For dental care
- To help reduce the effects of concussion
- Other:

Q.10. If you do not always wear a mouthguard, why not? Please rate the following answers: Strongly agree, agree, neutral, disagree, strongly disagree.

- Difficulty breathing
- Difficulty speaking
- Bad taste/odor
- Price
- Excess saliva production
- Dry mouth
- Nausea
- Discomfort
- Aesthetic reasons (don't like how they look)
- I don't think they're necessary for my sport
- Other:

Q.11. What do you feel is the function of the mouthguard in Gaelic football? Please rate the following answers: Strongly agree, agree, neutral, disagree, strongly disagree.

- Teeth protection
- Jaw protection
- Helps prevent or reduce the effects of a concussion
- Gum protection
- Cheek protection
- Other:

**Supplementary Material 3.** *Motivations and Barriers to Mouthguard Use Compliance (n = 545) Male Vs Female*

|  | Agree | | Neutral | | Disagree | |  |  |
| --- | --- | --- | --- | --- | --- | --- | --- | --- |
|  | Male % (n) | Female % (n) | Male % (n) | Female % (n) | Male % (n) | Female % (n) | Chi-Square (Φ) | P value |
| **Motivations** |  |  |  |  |  |  |  |  |
| *Rules of the Game* | 81% (167) | 88% (96) | 13% (27) | 11% (12) | 6% (12) | 0% (1) | 4.83 (0.12) | 0.09 |
| *Injury Insurance* | 75% (154) | 89% (97) | 16% (32) | 7% (8) | 10% (20) | 4% (4) | 8.99 (0.17) | 0.01* |
| *Dental Care* | 95% (196) | 91% (99) | 3% (7) | 8% (9) | 2% (3) | 0% (1) | 3.62 (0.11) | 0.16 |
| *Reduce Concussion Effects* | 30% (61) | 36% (39) | 45% (93) | 36% (39) | 25% (52) | 28% (31) | 2.62 (0.09) | 0.27 |
| **Barriers** |  |  |  |  |  |  |  |  |
| *Discomfort* | 83% (134) | 74% (51) | 8% (13) | 16% (11) | 9% (14) | 10% (7) | 3.50 (0.12) | 0.17 |
| *Difficulty Breathing* | 77% (124) | 84% (58) | 14% (22) | 14% (10) | 9% (15) | 2% (1) | 4.62 (0.14) | 0.10 |
| *Difficulty Speaking* | 88% (141) | 88% (61) | 8% (13) | 9% (6) | 4% (7) | 3% (2) | 0.29 (0.04) | 0.87 |
| *Bad Taste/Odour* | 26% (42) | 25% (17) | 26% (41) | 35% (24) | 48% (78) | 41% (28) | 2.17 (0.10) | 0.34 |
| *Cost* | 8% (12) | 4% (3) | 17% (27) | 28% (19) | 76% (122) | 68% (47) | 3.90 (0.13) | 0.14 |
| *Aesthetics* | 11% (17) | 9% (6) | 16% (25) | 22% (15) | 74% (119) | 70% (48) | 1.37 (0.08) | 0.51 |
| *Excess Saliva* | 43% (69) | 52% (36) | 21% (33) | 25% (17) | 37% (59) | 23% (16) | 3.98 (0.13) | 0.14 |
| *Dry Mouth* | 44% (71) | 41% (28) | 20% (32) | 33% (23) | 36% (58) | 26% (18) | 5.24 (0.15) | 0.07 |
| *Nausea* | 14% (22) | 19% (13) | 15% (24) | 33% (23) | 71% (115) | 48% (33) | 13.06 (0.24) | 0.00* |
| *Feel it is Unnecessary* | 12% (19) | 13% (9) | 21% (34) | 25% (17) | 67% (108) | 62% (43) | 0.50 (0.05) | 0.78 |
| ***Function*** |  |  |  |  |  |  |  |  |
| *Teeth Protection* | 99% (365) | 100% (178) | 1% (2) | 0% (0) | 0% (0) | 0% (0) | 0.97 (0.04) | 0.32 |
| *Jaw Protection* | 65% (240) | 68% (121) | 21% (76) | 25% (44) | 14% (51) | 7% (13) | 5.43 (0.10) | 0.07 |
| *Reduce Concussion Effects* | 37% (135) | 33% (59) | 40% (145) | 39% (69) | 23% (86) | 28% (50) | 1.50 (0.05) | 0.47 |
| *Gum Protection* | 65% (240) | 69% (122) | 23% (86) | 23% (40) | 11% (41) | 9% (16) | 0.77 (0.04) | 0.68 |

N: Number of participants; Φ: Phi coefficient effect size; *: significant p value < 0.05.

**Supplementary Material 4.** *Motivations and Barriers to Mouthguard Use Compliance (n = 545) Elite Vs Sub-Elite*

|  | Agree | | Neutral | | Disagree | |  |  |
| --- | --- | --- | --- | --- | --- | --- | --- | --- |
|  | Elite % (n) | Sub-Elite % (n) | Elite % (n) | Sub-Elite % (n) | Elite % (n) | Sub-Elite % (n) | Chi-Square (Φ) | P value |
| **Motivations** |  |  |  |  |  |  |  |  |
| *Rules of the Game* | 83% (29) | 84% (234) | 11% (4) | 13% (35) | 6% (2) | 39% (11) | 0.27 (0.03) | 0.87 |
| *Injury Insurance* | 69% (24) | 81% (227) | 17% (6) | 12% (34) | 14% (5) | 7% (19) | 3.52 (0.11) | 0.17 |
| *Dental Care* | 91% (32) | 94% (263) | 6% (2) | 5% (14) | 3% (1) | 1% (3) | 0.83 (0.05) | 0.66 |
| *Reduce Concussion Effects* | 31% (11) | 32% (89) | 29% (10) | 44% (122) | 40% (14) | 25% (69) | 4.46 (0.12) | 0.11 |
| **Barriers** |  |  |  |  |  |  |  |  |
| *Discomfort* | 78% (18) | 81% (167) | 13% (3) | 10% (21) | 9% (2) | 9% (19) | 0.19 (0.03) | 0.91 |
| *Difficulty Breathing* | 87% (20) | 78% (162) | 9% (2) | 15% (30) | 4% (1) | 7% (15) | 0.95 (0.06) | 0.62 |
| *Difficulty Speaking* | 91% (21) | 87% (181) | 4% (1) | 9% (18) | 4% (1) | 4% (8) | 0.52 (0.05) | 0.77 |
| *Bad Taste/Odour* | 30% (7) | 25% (52) | 13% (3) | 30% (62) | 57% (13) | 45% (93) | 2.93 (0.11) | 0.23 |
| *Cost* | 4% (1) | 7% (14) | 22% (5) | 20% (41) | 74% (17) | 73% (152) | 0.22 (0.03) | 0.89 |
| *Aesthetics* | 9% (2) | 10% (21) | 4% (1) | 19% (39) | 87% (20) | 71% (147) | 3.27 (0.12) | 0.20 |
| *Excess Saliva* | 44% (10) | 46% (95) | 30% (7) | 21% (43) | 26% (6) | 33% (69) | 1.25 (0.07) | 0.54 |
| *Dry Mouth* | 48% (11) | 43% (88) | 26% (6) | 24% (49) | 26% (6) | 34% (70) | 0.56 (0.05) | 0.76 |
| *Nausea* | 17% (4) | 15% (31) | 22% (5) | 20% (42) | 61% (14) | 65% (134) | 0.15 (0.03) | 0.93 |
| *Feel it is Unnecessary* | 17% (4) | 12% (24) | 35% (8) | 21% (43) | 48% (11) | 68% (140) | 3.64 (0.13) | 0.16 |
| ***Function*** |  |  |  |  |  |  |  |  |
| *Teeth Protection* | 100% (58) | 100% (485) | 0% (0) | 0% (2) | 0% (0) | 0% (0) | 0.24 (0.02) | 0.63 |
| *Jaw Protection* | 81% (47) | 65% (314) | 14% (8) | 23% (112) | 5% (3) | 13% (61) | 6.53 (0.11) | 0.04* |
| *Reduce Concussion Effects* | 43% (25) | 35% (169) | 41% (24) | 39% (190) | 16% (9) | 26% (127) | 3.41 (0.08) | 0.18 |
| *Gum Protection* | 79% (46) | 65% (316) | 9% (5) | 25% (121) | 12% (7) | 10% (50) | 7.69 (0.12) | 0.02* |

N: Number of participants; Φ: Phi coefficient effect size; *: significant p value < 0.05.

**Supplementary Material 5**

Data Repository - https://osf.io/kzwbt/
