# Supplementary material for: Motivators and barriers to mouthguard compliance by adult Gaelic football athletes
Source: PLoS One. 2025 Apr 23;20(4):e0315555. doi: 10.1371/journal.pone.0315555 (PMC12017504; doi:10.1371/journal.pone.0315555)
Supplement: Attachment — Submitted filename: Response to Reviewers.docx [file pone.0315555.s003.docx]

**Aoife Burke**

School of Health and Human Performance

FT206 Polaris Building

Dublin City University

Glasnevin

Dublin 9

aoife.burke@dcu.ie

21/02/2025

Dear Editor and Reviewers,

Firstly, the authors would like to thank you sincerely for taking the time and effort to provide us with your insight in reviewing our manuscript. We understand that this is completely voluntary, and would really like to express our gratitude. The authors have made some suggested changes within the manuscript, and have outlined those changes in addition to responses below.

**Reviewer 1:**

**Point 1:** This manuscript investigates the motivators and barriers to mouthguard compliance among adult Gaelic football players, which is of significant practical importance. The study employs a cross-sectional survey design with a large sample size, effectively capturing the current status of mouthguard use among players. The use of chi-square tests to analyze differences between groups is appropriate. The results reveal relatively low compliance rates, gender disparities, and key motivators and barriers, providing a basis for future improvements.

**Response to Point 1:** The authors would like to thank the Reviewer for their time and insight provided in reviewing this manuscript, it is very much appreciated.

**Changes from Point 1:** N/A

**Point 2:** However, there are several limitations in the study. Firstly, the survey did not collect information on the specific types of mouthguards used by the players. Different types of mouthguards (e.g., standard-fit, boil-and-bite, custom-fit) vary significantly in comfort and functionality, which could be a crucial factor influencing compliance. Secondly, the study did not inquire about players' history of dental injuries, which may substantially affect their motivation to wear mouthguards. Thirdly, the study focused solely on adult players and did not include adolescents or children, who also constitute a significant portion of Gaelic football participants and may have different compliance patterns. Lastly, relying solely on self-reported data from players may introduce subjective biases. It is recommended to incorporate observational methods to enhance the accuracy of the results.

**Response to Point 2:** Thank you for highlighting these limitations. The authors had outlined some of these limitations in their original manuscript, but have elaborated on these points so that the reviewer’s insights are more clearly stated.

**Changes from Point 2:** Lines 345-362: “There are three main limitations to this study. Firstly, the survey did not capture details on the type of mouthguard that participants were using at the time. Custom-fit, standard-fit and boil-and-bite mouthguards have been noted to vary significantly in comfort and in functionality. Knowing the type of mouthguard used may have provided further insight into why compliance was relatively poor in this study. Secondly, the survey did not establish if athletes had sustained a traumatic dental injury in the past, which could have substantial implications on future engagement with mouthguard compliance. It has been reported that mouthguard compliance improves in those with a history of injury, but this was not captured in this study. Thirdly, the main focus of this study was on adult Gaelic Football athletes, and did not capture the compliance of teenagers or children who make up a significant proportion of Gaelic Football athletes. Of note, mouthguard use only became compulsory in 2014 in the male game, and 2017 in the ladies game. At this time, the current participants of this study would have been teenagers and may not have been open to adapting their practices, especially if mouthguards were ill-fitting due to changes in maturation and growth occurring at a similar time. Due to ethical constraints, this study could only assess compliance in adults and thus future studies should assess compliance in adolescents and children. Perhaps compliance will improve as the years progress, especially where children who are currently participating will be encouraged to wear a mouthguard upon their first encounter with the sport. Lastly, this study employed self-reporting methods, which introduces a subjective bias.”

**Point 3:** Despite these limitations, the study provides valuable insights into mouthguard use in Gaelic football. The authors are encouraged to address the aforementioned limitations in future research by including data on mouthguard types, injury history, and compliance among younger players. Additionally, employing multiple research methods could reduce subjective biases. The study is worth publishing but would benefit from further refinement in subsequent research to enhance the comprehensiveness and reliability of the findings.

**Response to Point 3:** Thank you for this comment. The authors very much agree that further research is needed in this area, and a future research study is under ethical review to progress from the findings of this initial study. We have added a short paragraph at the end of the limitations section highlighting the areas that are clearly required to develop further and more robust understanding in the future. We hope that this is sufficiently captured.

**Changes from Point 3:** Lines 364-396: “Future studies should conduct spot-check observations of training sessions and games to more accurately determine compliance in both adult and minor populations, enhancing the accuracy of the results found in this study. Furthermore, details on the type of mouthguard owned by the athlete should be noted, alongside their history of dental injuries. These additional methodological enhancements would further develop our understanding of compliance and may potentially inform future policies and rules within the sport.”

**Reviewer 2:**

**Point 1:** This is an interesting and well-written study. To give more context to the study, it would have been interesting to mention how many people practice Gaelic football.

**Response t Point 1:** Thank you for your time and insight in reviewing this manuscript, your thoughts are very much appreciated. It is actually quite challenging to find an exact figure of Gaelic Football participation levels due to a lack of reporting by the governing body. However, based on a recent population consensus and a Sports Participation Report, we have estimated the number of adult participants who practice Gaelic Football at this time.

**Changes from Point 1:** Lines 65-66 “It is estimated that 27% of Ireland’s population participates in Gaelic Football , equating to roughly 472,500 adult players.”

**Point 2:** It would also add, if possible, a comparison with similar studies done for other sports are reported and if specific behavior could be identified for Gaelic football practitioners compared to other sports concerning mouthguard-wearing habits.

**Response to Point 2:** Thank you for your comment. In lines 100-102, the compliance in other sports is documented as part of the introduction (Across other team sports where mouthguard use is mandatory, compliance amongst adults has been found to be greatest in field hockey players (88-91%) [16,17], with lower rates observed in rugby players (54-68%) [18,19], and in ice hockey players (13-63%) [20,21].). This is then again addressed in the Discussion section at lines 264-266 (These compliance rates align well with previous research [27], which found compliance rates of 54% and 43% in rugby [22] and field hockey [28] respectively.). Following on from this, in lines 285-286, comparisons are made with regards to similar facilitators being observed in another study (This is in line with previous research whereby player perception of efficacy in injury prevention largely dictates implementation practices [18].), along with comparisons to studies assessing barriers to compliance also – lines 288-291 (A recently published review on sports mouthguards has identified similar barriers to compliance [32]. Although the mouthguard fit (e.g. custom-made vacuum fit mouthguards) can influence comfort and perceived ease of breathing and communication [33], this alone does not seem enough to command good compliance [12].).

With regards to specific behaviour, lines 296-302 have aimed to address this (Ultimately, education around mouthguard use appears to be the most advocated means of improving compliance [32,35], with lower compliance rates observed when education has not been provided [35]. The results of this study demonstrate a good understanding of mouthguard function amongst participants, especially with relation to dental, jaw and gum protection. Athletes seemed less sure of the potential benefits relating to concussion, and perhaps this is an avenue that should be explored more in player and coach education, especially if custom-fit mouthguards are implemented.)

**Changes from Point 2:** The authors are unsure if the reviewer would like more elaboration on the points noted perhaps? If this is the case, the authors will more than happily elaborate further on the comparisons made. The initial intention of this study was to provide a concise overview of an issue within Gaelic Football, so the authors did not write a lengthy discussion. However, we are certainly open to suggestion on this and will be guided by the views of the reviewer and Editor.

We hope that these changes are acceptable to you, and address your concerns appropriately. Please do not hesitate to contact me, should there be any queries or mis-understandings.

Many thanks in advance.

Best wishes,
